# Cardiac MR imaging and MR angiography in pediatric congenital heart disease: a comparison between 1.5T and 3.0T

**DOI:** 10.1186/1532-429X-15-S1-W27

**Published:** 2013-01-30

**Authors:** KL Nguyen, SN Khan, J Moriarty, K Mohajer, P Renella, G Satou, I Ayad, S Patel, I Boechat, P Finn

**Affiliations:** 1 Cardiology, UCLA, Los Angeles, CA, USA; 2 Radiology, UCLA, Los Angeles, CA, USA; 3 Pediatric Cardiology, UCLA, Los Angeles, CA, USA; 4 Anesthesia, UCLA, Los Angeles, CA, USA

## Background

To assess the feasibility of cardiac magnetic resonance imaging (MRI) and angiography (MRA) at 3.0T in pediatric patients with congenital heart disease (CHD) and to compare the technical and diagnostic performance with an age-matched and clinically comparable control group imaged at 1.5T.

## Methods

Forty-six patients (age 1 day to 8 years, mean 37.2 ± 32.8 months) with suspected or known CHD were evaluated (9/2008-5/2011). SSFP cine imaging, time-resolved MRA (TR-MRA), and high resolution contrast-enhanced MRA (HR-MRA) were performed. Two independent observers analyzed the MRI data for image quality, thoraco-abdominal vessel definition, and artifacts.

## Results

At 3.0T, 91% of SSFP cine images (k=0.55) and 97% (374 of 387) of vascular segments (k=0.49) were rated as good or excellent image quality with 72% of SSFP cine images having mild and 27% having moderate artifacts. At 1.5T, 92% of SSFP cine images (k=0.52) and 96% (344 of 356) of vascular segments (k=0.18) were rated as good or excellent image quality with 86% of SSFP cine images having mild and 14% having moderate artifacts. The SNR and CNR of SSFP images and HR-MRA were higher at 3.0T (p<0.001) with off-resonance artifacts being more prevalent at 3.0T (45% of images at 3.0T vs 27%). However, they rarely rendered the images non-diagnostic.

**Figure 1:**
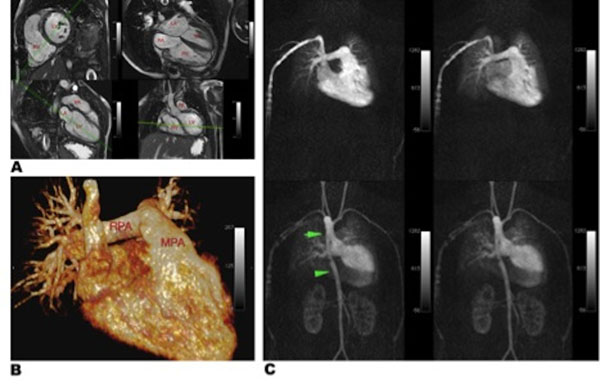
Six-year-old patient with Tetralogy of Fallot following complete repair with a monocuspid pulmonary valve, right ventricular outflow tract (RVOT) muscle resection and transannular patch placement, and free pulmonary regurgitation imaged at 1.5T. (A) SSFP cine images showing right ventricular hypertrophy and dilatation. (B) 3D volume rendered image showing enlarged main (22mm) and right (14mm) pulmonary arteries. (C) Time resolved CE-MRA demonstrates typical sequence of contrast opacification from a right peripheral vein and symmetric pulmonary parenchymal opacification without evidence of shunting (upper row). There is a right sided aortic arch with waisting of the descending aorta (lower row).

## Conclusions

Cardiac MRI & MRA at 3.0T are feasible in children with CHD. Both field strengths can be used successfully for cardiac and vascular imaging; deciding which to use depends on local availability and the importance of vascular (extra-cardiac) vs. intra-cardiac imaging.

## Funding

Siemens Research Grant

